# 1597. Randomized-Controlled Trial Evaluating The Outcome Between Switching to Tenofovir Disoproxil Fumarate(TDF)/Lamivudine(3TC)/Dolutegravir(DTG) Versus Maintaining The Current NNRTI Or Boosted-PI-containing Regimen in PLWH in Thailand – A Pilot Study from Single Center

**DOI:** 10.1093/ofid/ofad500.1432

**Published:** 2023-11-27

**Authors:** Samadhi Patamatamkul, Kongkarn Chancharusiri, Opass Putcharoen

**Affiliations:** Department of Medicine, Suddhavej Hospital, Mahasarakham University, Talat, Maha Sarakham, Thailand, 44000, Maha Sarakham, Maha Sarakham, Thailand; Department of Psychiatry, Suddhavej Hospital, Mahasarakham University, Talat, Maha Sarakham, Thailand, 44000, Maha Sarakham, Maha Sarakham, Thailand; Division of Infectious Disease, Department of Medicine, Faculty of Medicine, Chulalongkorn University, Krungthep, Krung Thep, Thailand

## Abstract

**Background:**

The Thai and WHO HIV 2022 guidelines have recently adopted TDF/3TC/DTG (TLD; single tablet) as the preferred regimen replacing NNRTI- or boosted-PI-based cART among both treatment-naïve and -experienced PLWH. Virologic and metabolic outcomes are scarce after switching to TLD compared with maintaining the same ART. Additionally, there are concerns regarding an increase in metabolic complications after switching to a DTG-based regimen, especially weight gain and possible subsequent metabolic syndrome. Previous data mostly consisted of the non-Asian population.

**Methods:**

We enrolled virologically suppressed PLWH age ≥18 years currently on NNRTI or boosted-PI-containing cART and randomized to either switching to TLD or maintaining their current cART during 2019-2021 at our tertiary care center. The primary outcome was virological suppression < 50 copies/mL at 48 weeks. Secondary outcomes were changes in CD4 counts, body weight, waist circumferences, metabolic profile, 9 questions depression rating scale (9Q), and sleeping quality measure by the Thai version of the Pittsburgh sleep quality index (T-PSQI) questionnaires at 48 weeks.

**Results:**

The study population comprised 48 persons; 23 were switched to TLD, and 25 continued their current cART. Seventy-eight and ninety-two percent of participants had an EFV-based regimen prior to switching in the TLD and cART group, respectively. The median age was 25 years and 80% were male. At 48 weeks, 19 and 20 in the TLD and current cART completed the follow-up. Baseline characteristics were shown in Table 1. Virological suppression was achieved in 100% vs. 90% in the TLD and current cART group, respectively (p=0.487). In addition to increasing sleeping quality, LDL and cholesterol significantly decreased after switching to TLD compared with the current ART (Figure 1). Waist circumference and BMI changes did not significantly differ between the two groups.

**Table 1.**
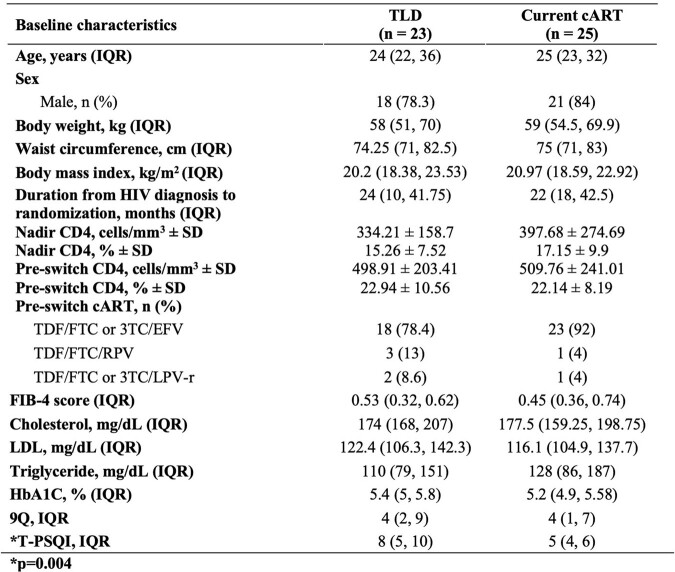
Baseline characteristics of all participants

The baseline characteristics of all participants demonstrated differences in the baseline T-PSQI in which the TLD group had a slightly higher median than the cART group. Asterisk represented a significant difference.

**Figure 1.**
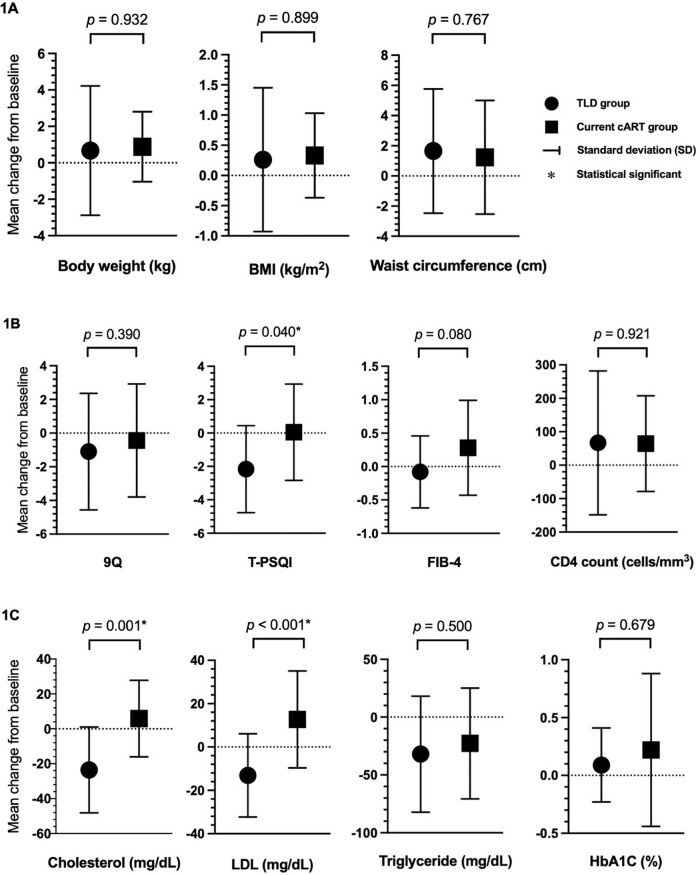
Mean changes of each parameter at 48 weeks post-switching

There was a significant reduction of cholesterol, LDL, and T-PSQI at 48-week post-switching in the TLD group than cART group. The lower T-PSQI represented better sleeping quality.

**Conclusion:**

Switching to TLD in PLWH maintained high virological suppression and achieved a favorable lipid profile and sleeping quality than maintaining NNRTI- or booster-PI-based regimen. Body weight and BMI changes were comparable and there was a trend toward a more favorable FIB-4 score in the TLD group. Our study supports the recommendation of switching to TLD among Thai PLWH.

**Disclosures:**

**All Authors**: No reported disclosures

